# Occurrence and Characterization of *Penicillium* Species Isolated from Post-Harvest Apples in Lebanon

**DOI:** 10.3390/toxins13100730

**Published:** 2021-10-16

**Authors:** Wassim Habib, Mario Masiello, Hala Chahine-Tsouvalakis, Zahraa Al Moussawi, Carine Saab, Salwa Tohmé Tawk, Luca Piemontese, Michele Solfrizzo, Antonio Francesco Logrieco, Antonio Moretti, Antonia Susca

**Affiliations:** 1 Laboratory of Mycology, Department of Plant Protection, Lebanese Agricultural Research Institute, P.O. Box 90-1965, Fanar 1202, Lebanon; whabib@lari.gov.lb (W.H.); carinesaab13@gmail.com (C.S.); 2 Faculty of Agricultural Sciences, Lebanese University, George Matta, Dekwaneh 1202, Lebanon; hala.chahine@gmail.com (H.C.-T.); zhraamoussawi@gmail.com (Z.A.M.); salwatawk@gmail.com (S.T.T.); 3 Institute of Science of Food Production—ISPA, Research National Council—CNR, Via Amendola, 122/O, 70126 Bari, Italy; michele.solfrizzo@ispa.cnr.it (M.S.); antonio.logrieco@ispa.cnr.it (A.F.L.); antonio.moretti@ispa.cnr.it (A.M.); antonella.susca@ispa.cnr.it (A.S.); 4 Department of Pharmacy and Pharmaceutical Sciences, University of Bari “Aldo Moro”, Via E. Orabona 4, 70125 Bari, Italy; luca.piemontese@uniba.it

**Keywords:** toxigenic fungi, patulin, beta tubulin, *Penicillium expansum*, *P. solitum*

## Abstract

The apple is one of the most important fruit tree crops in the Mediterranean region. Lebanon, in particular, is among the top apple producer countries in the Middle East; however, recently, several types of damage, particularly rot symptoms, have been detected on fruits in cold storage. This study aims to identify the causal agents of apple decay in Lebanese post-harvest facilities and characterize a set of 39 representative strains of the toxigenic fungus *Penicillium*. The results demonstrated that blue mould was the most frequent fungal disease associated with apples showing symptoms of decay after 3–4 months of storage at 0 °C, with an average frequency of 76.5% and 80.6% on cv. Red and cv. Golden Delicious apples, respectively. The morphological identification and phylogenetic analysis of *benA* gene showed that most *Penicillium* strains (87.2%) belong to *P. expansum* species whereas the remaining strains (12.8%) belong to *P. solitum*. Furthermore, 67.7% of *P. expansum* strains produced patulin when grown on apple puree for 14 days at 25 °C with values ranging from 10.7 mg kg^−1^ to 125.9 mg kg^−1^, whereas all *P. solitum* did not produce the mycotoxin. This study highlights the presence of *Penicillium* spp. and their related mycotoxin risk during apple storage and calls for the implementation of proper measures to decrease the risk of mycotoxin contamination of apple fruit products.

## 1. Introduction

Apples are one of the most cultivated and consumed fruits worldwide for their nutritional and organoleptic properties. Apples are rich in important bioactive molecules, such as antioxidants, flavonoids, and dietary fibres, which can ensure human health benefits, reducing the risk of hypertension, diabetes, heart disease and chronic diseases [1,2].

After harvest, apples can be stored in cold storage rooms for up to 12 months. However, during storage, several fungi can infect ripe fruits, such as *Botrytis cinerea* [3], *Venturia inequalis* [4] and fungal species belonging to *Botryosphaeria* [5], *Mucor* [6], and *Penicillium* genera. Two other pathogens have been also detected on apples in cold storage in the United States of America: *Sphaeropsis pyriputrescens* and *Phacidiopycnis washingtonensis* [7,8].

Among the post-harvest fungal diseases, blue mould decay is the most important worldwide. *Penicillium expansum* is the most common causal agent associated to blue mould on apples and pears; however, other *Penicillium* species, including *P. solitum*, *P. commune*, *P. verrucosum*, *P. chrysogenum*, *P. regulosum*, *P. crustosum* can occur on decayed apples and pears [9,10,11,12].

Punctures, bruises and cracks on fruit surface, caused at harvest or during the postharvest handling process, are the first cause of blue mould infections. In some particular conditions, blue mould may also originate from infections on the fruit’s pedicel and injured lenticels [10]. Decayed fruits have an earthy and musty smell; the infected area appears light tan to dark brown and tissues are soft and watery. The lesions have a very sharp margin between diseased and healthy tissues and blue or blue-green sporifications can appear on the surface of decayed tissues [10].

Blue mould disease is an economic concern, since it causes both productive and quality losses, and a serious risk for human health, because *P. expansum* and other *Penicillium* species associated to apple decay can synthetize toxic secondary metabolites. *Penicillium expansum* has a broad host range, including apple, pear, kiwi fruits, apricots, peaches, and strawberries. In pure culture, *P. expansum* is reported to produce several secondary metabolites [13]; however, patulin (PAT) is the main non-volatile compound produced. Patulin is a mycotoxin with potential mutagenic, carcinogenic, teratogenic and embryotoxic effects on humans [14] and represents a toxicological risk both to fresh fruit and fruit juice consumption. Indeed, PAT can be transferred into fruit juices if rotten fruits are not selectively discarded during fruit juice processing [15,16,17].

Apple trees are grown in Lebanon on a total area of 13,506 ha with a production of about 216,861 tonnes yearly, which constitute about 20% of total Lebanese fruits production [18]. The production capacity for high-quality apples in the country is insufficient to cover national needs because of the inefficient farming and bad post-harvest practices, such as over-application of pesticides, poor harvesting and handling practices, inappropriate transport methods, and below-standard cold storage conditions [19]. In terms of trade, the leading fruit export product in Lebanon is apples which constitute almost 25% of the total production [18]. The main destination market for Lebanese apples is Egypt with about 70% of all Lebanese apple exports and the rest is mainly exported to the Gulf states [20]. Nonetheless, unsold or discarded apples are processed mainly to produce juice and vinegar.

Recently, several types of damage, such as mechanical bruising, injuries, deformation, russetting, and rot have been detected on Lebanese apples in cold storage, which were attributed to bad harvesting and handling practices [21]. Apple production is one of the active fruit-growing sectors in Lebanon; several projects have been implemented in the last few years to reduce food loss during apple production and improve the competitiveness of apples in both domestic and international markets. The frequency of rotten apples in one crate can reach 4% in some packinghouses [21]; however, there is no available information regarding the causal agents of apple decay in Lebanese packinghouses, which makes this work original with regional interest. The aims of this study were, therefore, to evaluate the frequency of post-harvest decay diseases on apples in the packinghouses in Mount Lebanon, the major apple production area of the country, and to characterize, through morphological, molecular and chemical analyses, a set of representative *Penicillium* strains isolated from apples of that production area.

## 2. Results

### 2.1. Frequency of Post-Harvest Fungal Pathogens

Several fungal genera were recovered from decayed apple fruits collected from Lebanese cold packinghouse facilities after 3–4 months of storage at 0 °C (Table 1). The most common pathogens associated with post-harvest decay were fungi belonging to *Penicillium* genus, the causal agent of blue mould disease, with a frequency ranging from 37.5% to 89.5% (average 76.5%) and from 58.3% to 100% (average 80.6%) on Red and Golden Delicious apples, respectively. *Botrytis* spp., causal agent of gray mould, was the second most frequent post-harvest decay pathogen with an average frequency of 32.7% and 31.3% on Red and Golden Delicious apples, respectively. The packinghouse 1, located in Mayrouba, presented the highest frequency of blue mould (89.5% on Red Delicious, 100% on Golden Delicious) and the lowest frequency of gray mould (10.5% on Red Delicious, 0% on Golden Delicious), whereas packinghouse 5, located in Dbaye, harbored the highest frequency of gray mould (75% and 58.3% on Red and Golden Delicious, respectively) and the lowest frequency of blue mould (37.5% and 58.3% on Red and Golden Delicious, respectively). Furthermore, mixed infection by both pathogens was detected on 13.6% and 11.8% of Red and Golden Delicious sampled fruits, respectively.

Other post-harvest decay agents were also isolated from infected tissues with lower frequencies, namely *Alternaria* spp. (4.9% on Red Delicious, 2.1% on Golden Delicious), *Stemphylium* spp. (4.3% on Red Delicious, 3.5% on Golden Delicious), *Rhizopus* spp. (3.7% on Red Delicious, 1.4% on Golden Delicious), and *Cladosporium* (1.2% on Red Delicious, 1.4% on Golden Delicious). Sporadic occurrence (Incidence < 1%) of *Fusarium* spp., *Fusicoccum* spp., *Mucor* spp., and *Pestalotia* spp. was also identified on Red Delicious fruits.

### 2.2. Identification of Penicillium Isolates

The morphological identification showed that *Penicillium* strains from decayed apples from Mount Lebanon belong to two species: *P. expansum* and *P. solitum*. Several macro- and micro-morphological features on CYA, MEA and PDA culture media differed between the two species (Table 2).

In terms of colony characters, colonies of *P. expansum* are fasciculate on MEA presenting an orange brown to brown reverse colony color on CYA, whereas those of *P. solitum* present a velutinous texture and a light beige reverse color. On the other hand, the colony diameter of *P. expansum* isolates was larger than that of *P. solitum* on CYA (at 25 °C and 30 °C) and MEA after 7 days of incubation. Furthermore, synnemata were detected in almost all colonies of *P. expansum* on PDA after 1 month of incubation at 25 °C, whereas they were absent in *P. solitum* colonies.

In terms of micromorphological features observed in 7-day-old colonies on MEA, conidia of *P. solitum* and *P. expansum* differed in shape and size: *P. solitum* conidia are globose to sub-globose and are larger (5 × 4.7 µm) than the ellipsoid conidia of *P. expansum* (4.3 × 3.3 µm). Both species produce terverticillate conidiophores; however, the stipe is rough in *P. solitum* and smooth in *P. expansum*.

**Figure 1:**
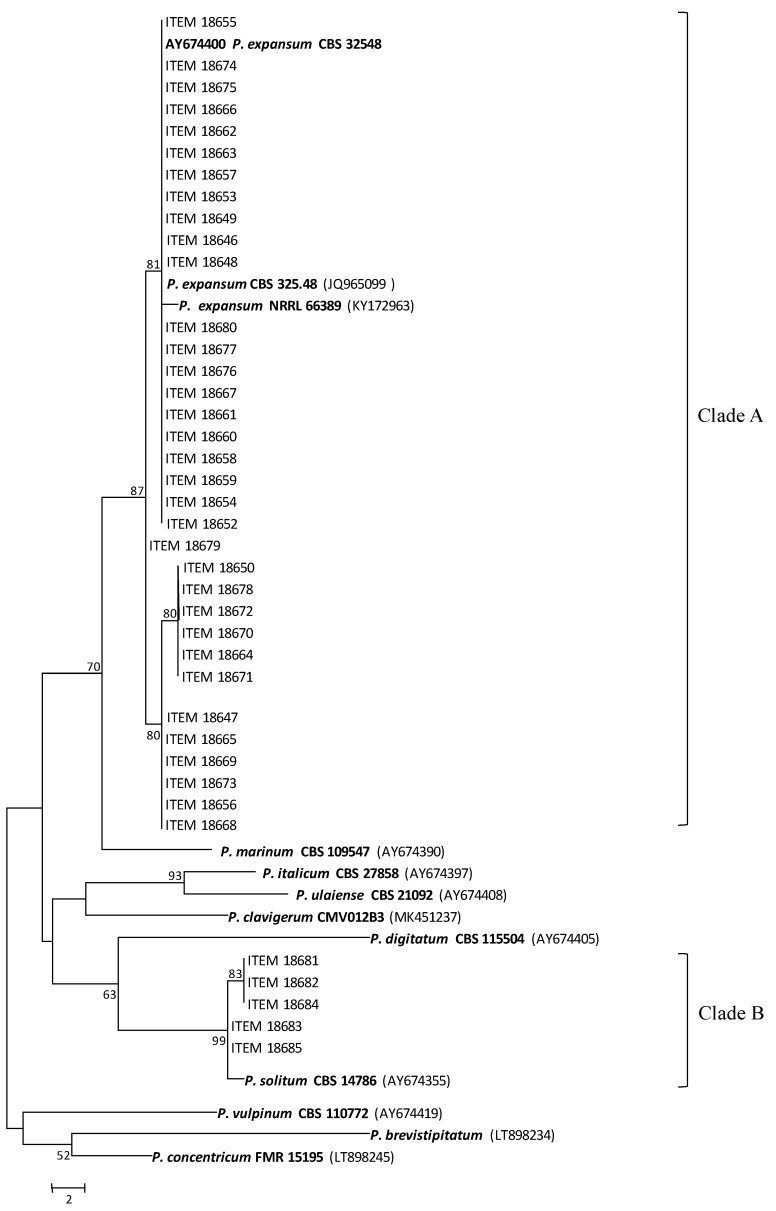
Phylogenetic tree inferred using the maximum parsimony method, based on the β-tubulin gene. The percentage of replicate trees in which the associated taxa clustered together in the bootstrap test (1000 replicates) are shown next to the branches. Bootstrap values > 50% (1000 replicates) are shown above or below the nodes.

Morphological identification was also supported by DNA-based identification. Indeed, 34 stains were identified as *P. expansum* and five as *P. solitum* by BLAST analysis, and confirmed by phylogenetic analysis of benA, which resolved the 39 strains in two well-separated clades (Figure 1). Tree #1 out of the six most parsimonious trees (length = 114) is shown. The consistency index is CI = 0.694444, the retention index is RI = 0.891089, and the composite index is 0.719125 for all sites and 0.618812 for parsimony-informative sites. Most of the strains (87.2%) were contained within a well-supported clade A (bootstrap value, 87), including three *P. expansum* reference species strains (CBS 32548, CBS 325.48 and NRRL 66389), and the remaining strains (12.8%) clustered with a *P. solitum* reference species strain (CBS 14786) within a well-supported clade B (bootstrap value, 99). GenBank accession numbers of the benA gene of the studied isolates are shown in Table 3.

### 2.3. Patulin Production In Vitro

Among the 39 *Penicillium* strains, 23 (59 %) produced patulin after 14 days of incubation on apple puree (Table 3). In particular, 67.7 % of *P. expansum* strains produced patulin with values ranging from 3.2 mg L^−1^ (LOD) to 125.9 mg L^−1^, whereas all *P. solitum* and 11 *P. expansum* strains did not produce the mycotoxin.

## 3. Discussion

In the food supply chain, significant productive and economic losses are caused by inadequate storage conditions as well as decisions made at the first processing stages, which predispose products to a shorter shelf life. In particular, inadequate storage facilities, poor handling practices, packaging and transportation are identified as the main critical loss points which can cause conspicuous losses [22].

Apples are prone to spoilage during transportation and storage, depending on a variety of internal and external factors that lead to infections by various diseases and pathogenic microorganisms, resulting in serious post-harvest losses, which have been reported in several countries, particularly in Lebanon [21].

The present study allowed to identify the fungal population associated to postharvest decay of cv. Red and Golden Delicious grown in Mount Lebanon. In addition to *Botrytis* spp., causing gray mould, the data generated revealed that the fungi most frequently occurring in our study were *P. expansum* and *P. solitum*, which cause blue mould disease. Both diseases have been reported as the dominant post-harvest diseases on pome fruits worldwide [23,24,25]. However, other fungal genera such as *Aspergillus*, *Phialophora*, *Pezicula*, *Colletotrichum*, *Mucor*, *Rhizopus*, *Alternaria*, *Botryosphaeria*, and *Fusarium* were also isolated from rotten pome fruits, according to previous reports [23,26]. Moreover, in specific studies, some *Neofabraea* spp. were the most prevalent fruit rot pathogens on apples in the Netherlands [27], while *Gloeosporium* spp. and *Monilinia fructigena* were considered as main pathogens in England and France [28].

*Penicillium* is one of the largest and most important toxigenic genera of microscopic fungi, with over 400 described species distributed worldwide [29]. Several *Penicillium* species, including *P. expansum*, *P. solitum*, *P. crustosum*, *P. italicum*, *P. digitatum*, *P. commune*, *P. verrucosum*, *P. rugulosum*, *P. polonicum*, *P. chrysogenum*, *P. allii* and *P. sumatraese* have been reported as responsible for apple decay worldwide [9,10,11,12,30]. In this study, a set of *Penicillium* strains isolated from decayed Red and Golden Delicious apples were identified and tested for their ability to produce mycotoxins. The molecular and morphological identifications led to the detection of two species: *P. expansum* (87.2%) and *P. solitum* (12.8%). Therefore, attention was focused on patulin, a well know mycotoxin produced by * P. expansum*.

Several studies have reported the toxic effect of patulin on humans and animals [31]. Indeed, the European Commission (1881/2006/EC, 2006), in order to protect consumers from unacceptable contamination, regulated the maximum levels of patulin permitted in certain foodstuffs, with a range that included between 50 μg kg^−1^ for fruit juices or nectars or spirit drinks and 10 μg kg^−1^ for drinks and food for infants and young children.

In the present study, around 70% of *P. expansum* strains produced patulin whereas none of *P. solitum* strains produced the mycotoxin. The data are consistent with previous reports, since *P. expansum* is considered as the major responsible of patulin occurrence in fruits and related products [32] and hence is a major concern in terms of food safety. Although *P. solitum* is unable to produce patulin, it acts as a predisposing agent, allowing an easier penetration of *P. expansum* in the apple tissues and, therefore, contributing to more destructive infections [33,34]. Recent genome sequencing of the two species has revealed an intact patulin gene cluster consisting of 15 biosynthetic genes identified in the patulin producing *P. expansum* strain R19, while only a remnant, seven-gene cluster in the patulin-deficient *P. solitum* strain. Nevertheless, *P. solitum* contained a large number of additional secondary metabolite gene clusters, indicating that this species has the potential capacity to produce an array of known as well as not-yet-identified products of possible toxicological or biotechnological interest [35].

The production of PAT depends on several factors, such as environmental conditions, apple cultivars and genetic diversity of *P. expansum* population. Susceptibility to *P. expansum* colonization and PAT accumulation differs among apple and pear varieties, suggesting that the content of acidity is one of the important factors affecting PAT levels [36,37,38], together with low temperature [39], which limits growth and PAT production of *P. expansum*.

Prevention of contamination of apples by PAT relies on the modulation of environment conditions during storage and application of good agricultural practices aiming at reducing the pathogen inoculum that could help maintain storage quality of apples and effectively reduce PAT in the human dietary route. In fact, several sources of *Penicillium* inoculum have been reported during the value chain of apples. In the orchard, *Penicillium* spp. is rarely present as airborne spores on the surface of fruits but is able to survive in organic debris and in soil [40,41]. In the packinghouse facility, flume water and dump-tank water are common sources of *Penicillium* spores, responsible for fruit infection during the handling and packing processes [10]. Spores of *Penicillium* are also commonly present in the air and on the walls of storage rooms [40]. During storage, *Penicillium* species can cause “nesting” in a fruit bin by growing into neighbouring healthy fruits [42] and they can represent secondary invaders of other rots. For these reasons, orchard and storage room’s sanitation and discarding rotten fruits and organic debris reduce inoculum sources of *Penicillium* spp., thus decreasing new infections on healthy fruits. Moreover, good pre- and post-harvest practices management, aiming at minimizing bruises on the fruit, prevents the infection by *Penicillium* species through wounds.

Ideally, detoxification could be also a solution to reduce PAT contamination. The applied PAT detoxification procedures should not only reduce the content of toxins to safe levels, but also prevent the reduction of the nutritional and palatable values of the treated commodity. Microorganisms have been always considered as promising candidates in reducing the exposure to mycotoxins from infected food crops and fruits. Indeed, some biocontrol agents are able to adsorb mycotoxins from an aqueous solution, and others can even transform the mycotoxin into less toxic or non-toxic products [43]. For instance, an atoxigenic strain of *Aspergillus flavus* is capable of reducing the patulin produced by the toxigenic *A. terreus* [44], and some enzymes are recognized as promising biocontrol agents in patulin detoxification [45]. Nevertheless, prevention of contamination still remains the most effective effort against patulin contamination of apple and processed apple-based products.

Although the results obtained refer to samples collected in 2016, the results of the present study highlight that the prevalent presence of *P. expansum* on apples during apple storage, with its related mycotoxin risk, is a reason for higher concern about the apple by-products. Therefore, the implementation of control strategies and proper measures in cold storage to protect fruits from fungal decay and increase food safety by decreasing the risk of mycotoxin contamination of apple fruit products is confirmed also in Lebanon. Indeed, in this country the prevailing environmental conditions can multiply the risks of post-harvest contamination of apple fruits. Also, the high percentage of strains able to produce patulin (around 70%) shows how extended is the risk of high contamination of the apples intended for processing. Finally, a further reason of concern can be related to the high genetic variability detected among the population of *P. expansum* studied, since three clearly distinct clades occurred in the phylogenetic tree. A high genetic variability can be a precious source of recombination for the fungus, aimed to select more aggressive pathogenic strains or strains provided with a higher ability to produce patulin.

## 4. Materials and Methods

### 4.1. Apple Sampling

Sampling was conducted in Kesserwan district (Mount Lebanon governorate) in February 2016 by randomly collecting 306 fruit samples showing symptoms of decay (162 from ‘Red Delicious’ and 144 from ‘Golden Delicious’ varieties), from five packinghouse facilities, which were sorting and grading apples stored for 3–4 months at 0 °C with 90% relative humidity, before market distribution. In each packinghouse, all apple fruits discarded during sorting and grading for a period of 3 h were gathered, and fruits showing initial symptoms of rot were considered for mycological analysis.

### 4.2. Isolation of Fungi

The isolation of fungi from the surface or the inner part of decayed apples was carried out by first decontaminating the fruits through immersion in 70% ethanol solution for 1 min and washing twice with sterile distilled water before the removal of the skin. Then, pieces of tissue (5 × 5 mm) taken from the margin of the infected areas were transferred onto potato dextrose agar (PDA, Himedia, India) medium added with 0.5 g L^−1^ streptomycin sulfate. Plates were incubated at 22 °C for 5–7 days in dark and pure cultures of recovered fungal isolates were obtained and identified based on their cultural and microscopic features [46].

The incidence of infection by each fungal pathogen was determined in each packinghouse and variety by calculating the percentage of fruits infected by a pathogen over the total of decayed fruits.

### 4.3. Characterization of Penicillium Isolates

Thirty-nine representative monosporic isolates of *Penicillium*, obtained by spreading conidial suspensions on 2.5% water agar and collecting single germinating spores under the stereomicroscope after 16–18 h incubation at 22 °C, were selected for further morphological and molecular identification, as well as for evaluating their ability to produce patulin in vitro.

#### 4.3.1. Morphological Characterization

The morphological characterization of *Penicillium* isolates were carried out according to Frisvad and Samson [47,48] and Visagie et al. [29]. Isolates were grown on Malt Extract Agar (MEA, Samson et al. 2010) and PDA at 25 °C in the dark, and on Czapeck Yeast Autolysate agar (CYA) [49] at 25 °C, 30 °C and 37 °C, to assess several macro and micro-morphological features. The macro-morphological characteristics assessed were the colony growth, color, and texture, in addition to the presence of exudates and synnemata (three replicates per strain). Microscopic slides were prepared from MEA in 60% lactic acid added as a mounting fluid to observe conidia size, conidia ornamentation, conidia color, stipe ornamentation, conidiophores pattern. The conidial measurements were taken from five replicates for each strain at 40× magnification

#### 4.3.2. Molecular Identification

For mycelium production, a suspension of spores from each fungal strain was grown in Wickerham medium (40 g of glucose, 5 g of peptone, 3 g of yeast extract, 3 g of malt extract and water up to 1 L), in darkness at 25 ± 1 °C on an orbital shaker (150 rpm) for 48 h. Mycelia were filtered, lyophilized and ground using a 5 mm iron bead in a Mixer Mill MM 400 (Retsch GmbH, Haan, Germany). DNA was isolated using “Wizard^®^ Magnetic DNA Purification System for Food” kit (Promega, Madison, WI, USA), with 10 mg of lyophilized mycelium as starting material. The quality of genomic DNA was determined by electrophoresis on 0.8% agarose gel and quantification by ND-1000 NanoDrop spectrophotometer (Thermo Fisher Scientific, Waltham, MA, USA).

In order to identify *Penicillium* species, the partial beta-tubulin gene (benA, ca. 450 nt) was selected since it has been recommended as an excellent marker to delineate the species of this genus [29]. The benA gene was amplified using primers (BT2a/BT2b) and related PCR conditions described in literature [50]. PCR products were purified with the enzymatic mixture EXO/SAP (Exonuclease I, *Escherichia coli*/Shrimp Alkaline Phosphatase) and processed for bidirectional sequencing. Sequence reactions were performed with the BigDye™ Terminator v3.1 Cycle Sequencing Kit (Thermo Fisher Scientific) for both strands, purified by gel filtration through Sephadex G-50 (Amersham Pharmacia Biotech, Buckinghamshire, UK) and analyzed on the “ABI PRISM 3730 Genetic Analyzer” (Applied Biosystems, San Francisco, CA, USA). The alignment of the two strands for each locus was performed using the software package BioNumerics 5.1 (Applied Maths, Sint-Martens-Latem, Belgium), with manual adjustments where necessary.

Species identifications were performed for each strain using the BLAST algorithm (http://www.ncbi.nlm.nih.gov, accessed on 10 September 2020), comparing beta tubulin sequences against the non-redundant database maintained by the National Center for Biotechnology Information.

To infer phylogenetic relationships, nucleotide sequences of the housekeeping gene (benA) were aligned with ClustalW and then analysed using the maximum parsimony (MP) method, with MEGA version 7.0 [51], including type- or reference-species strains. All positions containing gaps and missing data were eliminated from the analyses. Statistical significance of branches in the resulting tree was assessed by bootstrap analysis using 1000 pseudoreplications [52]. Branches of zero length were collapsed and all multiple, equally parsimonious trees were saved. The bootstrap consensus tree inferred from 1000 replicates was taken to represent the evolutionary history of the taxa analyzed. Bootstrap values > 50% (1000 replicates) are shown above or below the nodes. All positions containing gaps and missing data were eliminated from the dataset. *Penicillium vulpinum*, *P. brevistipatum* and *P. concentricum*, belonging to Clade 6 (Section: *Robsamsonia* according to Houbraken et al. [53] were included as outgroup. *Penicillium marinum*, *P. digitatum*, *P. italicum*, *P. clavigerum* and *P. ulaiense* were included because members of the same *Penicillium* Clade as *P. expansum* (Clade 2: *Penicillium*, according to Houbraken et al. [53].

### 4.4. Patulin Determination In Vitro

Apple puree cv. Golden Delicious was obtained from 2 kg of apples and 100 mL were distributed in 500 mL flasks, and then sterilized in an autoclave at 121 °C for 10 min. Flasks were then inoculated with 1 mL spore suspensions (10^6^ spores/mL) collected from single pure cultures of each strain and incubated at 25 °C in static conditions for 14 days. After incubation, 100 mL of ultrapure water were added to each flask and stirred for 1 h. When necessary, cultures were homogenized with a blender.

For patulin analysis, cultures were centrifuged at 386× *g* for 10 min and 1 mL of supernatant was diluted with 9 mL of ultrapure water and then 2 mL of this solution (equivalent to 2.5 µL of fungal culture) were filtered on PTFE syringe filters (0.45 µm). Then, 50 µL of the filtered solution were injected into a HPLC-UV (DAD) Agilent 1260 Infinity system equipped with a G1312 binary pump, a G1367E autosampler with a 100 μL loop, a G1316C column thermostat set at 30 °C, a G4212B UV (DAD) detector set at λ = 276 nm, and a software for Microsoft Windows 7 (Open LAB, CSD, Chemstation Edition). The separations were performed with a C18 Luna^®^ analytical column, 150 × 4.6 mm, 5 µm, 100 Å (Phenomenex, Torrance, CA, USA), preceded by a 3 mm, 0.45 μm pore size guard filter (Rheodyne, Cotati, CA, USA). The mobile phase was a 10% aqueous acetonitrile solution eluted at a flow rate of 1 mL/min. The detection limit (LOD) and the quantification limit (LOQ), calculated as S/N = 3 and S/N = 6, were 3.2 mg L^−1^ and 6.4 mg L^−1^, respectively. Patulin was identified by comparing the UV spectrum and the retention time of the peak recorded in the chromatogram of each sample with those of an authentic standard. UV spectra were recorded in the range of 200–400 nm. The quantitation of the toxin was performed according to an external standard method. The standard solution of patulin (100 µg/mL in acetonitrile) was purchased from Romer Labs Division Holding GmbH (Getzersdorf, Austria). The different calibration solutions of patulin were prepared by appropriately diluting aliquots of the commercial standard solution with HPLC mobile phase.

## Figures and Tables

**Table 1:** Frequency of blue mould and gray mould caused by *Penicillium* and *Botrytis*, respectively on decayed apples from five packinghouse facilities in Mount Lebanon.

Facility	Location	‘Red Delicious’			‘Golden Delicious’		
		Decayed Fruits (No.)	Blue Mould (%)	Gray Mould (%)	Decayed Fruits (No.)	Blue Mould (%)	Gray Mould (%)
1	Mayrouba	38	89.5	10.5	24	100	0
2	Hrajel	39	74.4	48.7	30	86.7	26.7
3	Hrajel	37	83.8	10.8	33	87.9	15.2
4	Kfardebian	40	67.5	50.0	33	69.7	54.5
5	Dbaye	8	37.5	75.0	24	58.3	58.3
Total		162	76.5	32.7	144	80.6	31.3

**Table 2:** Comparison between the morphological characteristics of *Penicillium expansum* and *P. solitum* strains isolated from decayed apples in Mount Lebanon.

Morphological Characteristics	*P. expansum*	*P. solitum*
*Characteristics on CYA (7 days)*		
Colony diameter at 25 °C (mm)	29–47 (x = 37.9) ^a^	21–29 (x = 26.1) ^a^
Colony diameter at 30 °C (mm)	6–13 (x = 10)	0–5.5 (x = 2.6)
Colony diameter at 37 °C (mm)	0	0
Color obverse	Dull green to green	Blue green to dark green with white margin
Color reverse	Orange brown to brown	Light beige
Exudate presence	None	None
*Characteristics on MEA (7 days at 25 °C)*		
Colony diameter (mm)	27–39 (x = 34.4)	22–25 (x = 23.3)
Colony texture	Fasciculate	Velutinous, floccose in center
Color colony obverse	Dull green to green	Blue green to dark green
Conidia shape	Ellipsoid	Globose to sub-globose
Conidia Length × width (µm)	4.2–4.4 × 3.2–3.4 (x = 4.3 × 3.3)	4.6–5.0 × 4.3–4.8 (x = 5.0 × 4.7)
Conidia ornamentation (wall)	Smooth	Smooth
Conidia color	Green	Green
Stipe ornamentation	Smooth	Rough
Conidiophore pattern	Terverticillate	Terverticillate
*Characteristics on PDA (30 days at 25 °C)*		
Synnemata presence	Often present	None

^a^ Measurements and observations were undertaken on 34 isolates of *P. expansum* and 5 isolates of *P. solitum*.

**Table 3:** *Penicillium* isolates from decayed apples in Mount Lebanon: origin, species, GenBank accession number of benA gene sequence, and patulin production after 14 days of incubation on apple puree.

*Penicillium* Species	Strain Number	Origin		Accession Number	Patulin (mg L^−1^) ^a^
		Town	Apple Cultivar		
*P. expansum*	ITEM 18646	Mayrouba	Red Delicious	FR997369	95.2
	ITEM 18647	Mayrouba	Red Delicious	FR997367	n.d.
	ITEM 18648	Mayrouba	Red Delicious	FR997368	46.2
	ITEM 18649	Mayrouba	Golden Delicious	FR997370	125.9
	ITEM 18650	Mayrouba	Golden Delicious	FR997371	40.6
	ITEM 18652	Hrajel	Red Delicious	FR997374	<LOQ
	ITEM 18653	Hrajel	Red Delicious	FR997375	n.d.
	ITEM 18654	Hrajel	Red Delicious	FR997376	n.d.
	ITEM 18655	Hrajel	Red Delicious	FR997372	10.7
	ITEM 18656	Hrajel	Red Delicious	FR997373	101.0
	ITEM 18657	Hrajel	Golden Delicious	FR997378	n.d.
	ITEM 18658	Hrajel	Golden Delicious	FR997379	n.d.
	ITEM 18659	Hrajel	Golden Delicious	FR997377	n.d.
	ITEM 18660	Hrajel	Red Delicious	FR997381	n.d.
	ITEM 18661	Hrajel	Red Delicious	FR997383	n.d.
	ITEM 18662	Hrajel	Red Delicious	FR997384	34.7
	ITEM 18663	Hrajel	Red Delicious	FR997380	<LOQ
	ITEM 18664	Hrajel	Red Delicious	FR997382	24.8
	ITEM 18665	Hrajel	Golden Delicious	FR997385	75.3
	ITEM 18666	Hrajel	Golden Delicious	FR997386	n.d.
	ITEM 18667	Hrajel	Golden Delicious	FR997387	13.5
	ITEM 18668	Hrajel	Golden Delicious	FR997388	47.9
	ITEM 18669	Kfardebian	Red Delicious	FR997389	83.6
	ITEM 18670	Kfardebian	Red Delicious	FR997390	16.3
	ITEM 18671	Kfardebian	Red Delicious	FR997391	n.d.
	ITEM 18672	Kfardebian	Golden Delicious	FR997394	91.7
	ITEM 18673	Kfardebian	Golden Delicious	FR997395	26.4
	ITEM 18674	Kfardebian	Golden Delicious	FR997396	19.7
	ITEM 18675	Kfardebian	Golden Delicious	FR997392	23.8
	ITEM 18676	Kfardebian	Golden Delicious	FR997393	n.d.
	ITEM 18677	Dbaye	Red Delicious	FR997397	<LOQ
	ITEM 18678	Dbaye	Red Delicious	FR997398	31.3
	ITEM 18679	Dbaye	Golden Delicious	FR997400	13.8
	ITEM 18680	Dbaye	Golden Delicious	FR997399	<LOQ
*P. solitum*	ITEM 18681	Mayrouba	Red Delicious	FR997401	n.d.
	ITEM 18682	Hrajel	Red Delicious	FR997402	n.d.
	ITEM 18683	Hrajel	Golden Delicious	FR997403	n.d.
	ITEM 18684	Hrajel	Golden Delicious	FR997405	n.d.
	ITEM 18685	Hrajel	Golden Delicious	FR997404	n.d.

^a^ Detection Limit (LOD) = 3.2 mg L^−1^ (S/N = 3), Quantification Limit (LOQ) = 10.7 mg L^−1^ (S/N = 10), n.d. = not detected.

## Data Availability

The data presented in this study are available within the article.
